# Lidocaine inhibits the metastatic potential of ovarian cancer by blocking Na_V_1.5‐mediated EMT and FAK/Paxillin signaling pathway

**DOI:** 10.1002/cam4.3621

**Published:** 2020-12-06

**Authors:** Chang Liu, Ming Yu, Yi Li, Hao Wang, Chuanya Xu, Xiaoqing Zhang, Min Li, Hongyan Guo, Daqing Ma, Xiangyang Guo

**Affiliations:** ^1^ Department of Anesthesiology Peking University Third Hospital Beijing China; ^2^ Department of Biochemistry and Molecular Biology Dalian Medical University Dalian China; ^3^ Department of Obstetrics and Gynecology Peking University Third Hospital Beijing China; ^4^ Anaesthetics, Pain Medicine and Intensive Care Department of Surgery and Cancer Faculty of Medicine Imperial College London Chelsea and Westminster Hospital London United Kingdom

**Keywords:** cisplatin, lidocaine, metastasis, Na_V_1.5, ovarian cancer

## Abstract

Lidocaine, one of the most commonly used local anesthetics during surgery, has been reported to suppress cancer cell growth via blocking voltage‐gated sodium channels (VGSCs). VGSC 1.5 (Na_V_1.5) is highly expressed in invasive cancers including ovarian cancer. This study aims to investigate whether lidocaine inhibits the malignancy of ovarian cancer through Na_V_1.5 blockage. Human ovarian cancer, its metastatic cancer and normal ovarian tissues were probed with anti‐Na_V_1.5 antibody *in situ*. Human ovarian cancer A2780 and SKOV3 cells were cultured and their growth, epithelial‐mesenchymal transition (EMT), migration, and invasion in the presence or absence of lidocaine together with underlying molecular mechanisms were assessed. Murine syngeneic ovarian cancer (ID8) model was also used to determine the chemotherapeutic efficiency of cisplatin in combination with lidocaine. The high level of Na_V_1.5 expression was found in human ovarian cancer and even higher in its metastatic cancer but not in normal ovarian tissues. Lidocaine decreased the growth, EMT, migration, and invasion of human ovarian cancer A2780 and SKOV3 cells. Lidocaine enhanced the chemotherapeutic efficiency of cisplatin in both ovarian cancer cell cultures and a murine ovarian metastatic model. Furthermore, a downregulation of Na_V_1.5 by siRNA transfection, or FAK inhibitor application, inhibited the malignant properties of SKOV3 cells through inactivating FAK/Paxillin signaling pathway. Our data may indicate that lidocaine suppresses the metastasis of ovarian cancer and sensitizes cisplatin through blocking Na_V_1.5‐mediated EMT and FAK/paxillin signaling pathway. The translational value of lidocaine local application as an ovarian cancer adjuvant treatment warrants further study.

## Graphical abstract

Local anesthetic lidocaine suppresses the metastasis of ovarian cancer and sensitizes cisplatin through blocking Na_V_1.5‐mediated EMT and FAK/paxillin signaling pathway. The translational value of lidocaine local application as an ovarian cancer adjuvant treatment warrants further study.

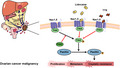

## 1 INTRODUCTION

Ovarian cancer is one of the malignant gynecological cancers with the highest mortality rate.^1^ When diagnosed, the majority of the patients are on the line of advanced or late stage. Ovarian cancer has strong metastatic capability and can spread to be abdominal transcoelomic metastasis rapidly and widely; this feature is also associated with the poor prognosis, and up to 80% of recurrence can occur after surgery within 18 months.^2^, ^3^ The main strategy of ovarian cancer treatment is to resect the primary cancer or to cytoreduct the recurred metastatic cancer loci together with the chemotherapy.^4^ However, the accumulative data revealed that surgical procedures may lead to systemic stress, inflammation, neuroendocrine responses, and immunosuppression, all of which not only facilitates the residual or micrometastatic foci dissemination perioperatively, but also potentiates cancer metastasis and recurrence after surgery.^5^ Current data suggest that anesthetics or anesthetic techniques may also affect the cancer metastasis and recurrence after surgery.^6^, ^7^ However, this field of research just starts and more studies are urgently needed.

Lidocaine is an amide local anesthetic, and widely applied in various surgeries including gynecological operations. It can effectively attenuate the postoperative pain, and reduce the dose and side effects of opioids.^8^ Recent studies also suggest that lidocaine has anticancer potential,^9^, ^10^ but underlying mechanisms remain unknown fully.

Lidocaine blocks voltage‐gated sodium channels (VGSCs) and causes the depolarization of excitable cells.^11^ VGSCs consist of α‐ and β‐subunits and nine α‐subunits (Na_V_1.1–1.9) have been identified; it has been considered that α‐subunit is correlated with the tempospacial and physiopathological specificities.^12^ Sodium ion channels are generally expressed in the excitable cells, such as nerve, myocardium, and skeletal muscle cells.^13^ However, recent studies reveal that many metastatic cancers also show abnormally high level expression of VGSCs and are closely correlated with cancer clinical staging, recurrence, drug resistance, and prognosis.^14^ Previous studies even indicated that specific Na_V_1.5 coded by *SCN5A* gene was increased and associated with the proliferation and metastasis of breast cancer and colon cancer cells.^15^, ^16^ Giving that lidocaine is often used in ovarian cancer surgery, this study aimed to investigate whether lidocaine could inhibit the malignancy of ovarian cancer through blocking Na_V_1.5 and the associated mechanisms.

## 2 MATERIALS AND METHODS

### 2.1 Tissue samples

Human normal ovarian tissue slides (OV806, 30 cases) and cancerous ovarian tissue slides (OV8010, 71 cases of stage II–III) were obtained from Alenabio (Xian, China). After ethic approval was given by both the Second Affiliated Hospital of Dalian Medical University and Peking University Third Hospital, 16 paired patient samples of both primary ovarian cancer and the metastatic lesions (omentum majus, colon, and vermix) were obtained from the Second Affiliated Hospital of Dalian Medical University (Supplemental Table S1). All tissue slides were stained for immunofluorescence of Na_V_1.5 with anti‐Na_V_1.5 antibody (Abcam, Cambridge MA, USA).

### 2.2 Cell culture

Human ovarian cancer A2780 and SKOV3 cells (ATCC, Manassas, USA) were cultured in RPMI 1640 (Hyclone, Utah, USA) and McCoy's 5A (Hyclone, Utah, USA) medium supplemented with 10% of fetal bovine serum (FBS) (Gibco, USA), 100 U/ml of penicillin, and 100 μg/ml of streptomycin at 37°C in humidified air containing 5% of CO_2_ incubator, respectively. The medium was replaced every 2–3 days. When cells reached to 90% confluence, they were treated with lidocaine (Tiansheng Pharmaceutical Group, Hubei, China) and cisplatin (Northeast Pharmaceutical Group, Shenyang, China), respectively in the medium for up to 48 hours for further experimental analyses.

### 2.3 Cell viability assay

Cells (5,000/well) seeded in 96‐well plate were treated with lidocaine (0, 1, 2.5, 5, 7.5, and 10 mM) and cisplatin (10 μM), respectively. Then, Cell Counting Kit‐8 (CCK‐8) reagent (Dojindo Molecular Technologies, Japan) was added to the well, followed by incubation at 37°C for 2 hours. The absorbance at 450 nm was measured using a microplate reader (Thermo Fisher Scientific, USA). Each assay was conducted in triplicate.

### 2.4 EdU incorporation assay

Incorporation assay (RIBOBIO, Guangzhou, China) was conducted with the manufacturer's instructions. Briefly, cells were incubated with 5‐ethynyl‐2′‐deoxyuridine (EdU) ‐labeling solution at 37°C for 3 hours, and then, fixed with 4% of paraformaldehyde for 30 min. After permeabilization in 0.5% of Triton X‐100, cells were kept in Apollo^®^ reaction solution for 30 min. Hoechst 33342 was used for nuclei staining, followed by photography under fluorescent microscope (Olympus, Japan).

### 2.5 Real‐time quantification PCR

Total RNA was extracted with RNAiso Plus reagent (Takara, Liaoning, China), and cDNA was synthesized using PrimeScript RT reagent Kit with a gDNA Eraser kit (Takara). The primers of qRT‐PCR are listed in the Supplemental Table S2. Cycle amplifications were completed by applying Applied Biosystems 7500 Fast Real‐time PCR System (Life Technologies, USA). Quantified data were normalized to those of GAPDH, and the relative quantity was calculated using the 2−ΔΔ^CT^ method.

### 2.6 Western blot

Proteins extracted from cell lysates were electrophoresed in 10% of SDS‐PAGE gel, and transferred onto a nitrocellulose membrane. After blocking with 5% of defat milk for 2 hours, membranes were incubated with the primary antibody PCNA (#10205), Cyclin D1(#26939), Cyclin E1 (#11554), E‐cadherin (#20874), N‐cadherin (#22018), Vimentin (#10366), PARP (#13371), Caspase‐3 (#19677), Caspase‐8 (#13423), and Bcl‐2 (#12789) purchased from Proteintech (Wuhan, China); Cleaved‐caspase‐3 (#9664), FAK (#3285), p‐FAK (#8556), Paxillin (#12065), and p‐Paxillin (#69363) purchased from Cell Signaling Technology (Boston, USA) at 4°C overnight. The antibody was properly diluted (Cleaved‐caspase‐3, p‐FAK, and p‐Paxillin in 1:500 and others in 1:1000). Then, membranes were incubated with HRP‐labeled goat anti‐rabbit or anti‐mouse IgG for 1 hour. An enhanced chemiluminescence (ECL) detection system (Bio‐Rad, USA) was used to visualize immunoreactive bands.

### 2.7 Immunofluorescent and immunohistochemical staining

Slides of human ovarian tissues were deparaffinized and rehydrated routinely. Endogenous peroxidase activity was inactivated by incubation in 0.3% of hydrogen peroxide for 15 min. Cells on the coverslips were fixed in 4% of paraformaldehyde for 30 min. After blocking with 10% of goat serum, tissues or cells were then incubated with the primary antibody at 4°C overnight, followed by adding FITC (green) or TRITC (red)‐conjugated second antibody for 1 hour. DAPI (blue) was used for nuclei staining for 5 min. Then, anti‐fade solution was dropped onto the slides or coverslips, followed by photography under fluorescent microscope. For immunohistochemical staining of the murine ovarian tissues, slides were pretreated similarly to those of the human ovarian tissue slides. Then, slides were incubated with the primary antibody at 4°C overnight, followed by incubation with biotinylated secondary antibody for 20 min. The coloration was got by binding of streptavidin‐peroxidase conjugate and chromogenic substrate (DAB). Mayer's hematoxylin was used as a counterstained dye. Images were captured with the microscope. The slides were examined by an independent clinical pathologist and two other experienced researchers who were blinded to research protocols. The Image J software (JAVA) was used for quantification.

### 2.8 Scratch assay

Cells in 90% confluence were scratched with a pipette tip. After brief wash with culture medium, cells were treated with 5 mM of lidocaine or untreated as a control. Wounded cultures were incubated in the incubator for 36 hours. Subsequently, three random fields at the lesion border were observed and photographed under inverted phase contrast microscope.

### 2.9 Transwell migration and matrigel invasion assays

Cell migration and invasion potential were assessed by transwell kits (Corning, Tewksbury, USA). Cells (50,000/well) were cultured FBS‐free medium for 12 hours on inserts placing in the upper chamber either with non‐coated membrane for migration assay, or with matrigel‐coated membrane for invasion assay. In the lower chamber, 600 μl of culture media RPMI 1640 supplemented with 10% of FBS for cell line A2780, and culture media McCoy's 5A supplemented with 10% of FBS for cell line SKOV3. After incubation for 12 hours (migration) or 18 hours (invasion), cells at the bottom side of the inserts were fixed with 100% of methanol for 20 min, followed by staining with 0.1% of crystal violet for 15 min. Images were captured with the microscope. Each assay was conducted in triplicate.

### 2.10 Gelatin zymography

The supernatants of the cell culture were electrophoresed in 10% of SDS‐PAGE gel copolymerized with 1% of gelatin. After electrophoresis, the gel was washed in 2.5% of Triton X‐100 for 1 hour, and then, incubated in 50 mM of Tris‐HCl, pH 7.6, and 5 mM of CaCl_2_ at 37°C for 18 hours. The gel was stained in 0.1% of Coomassie blue R250 solution for 2 hours, followed by distained in 10% of methanol and 10% of acetic acid in H_2_O. The transparent bands on the blue gel represent MMP‐2 and MMP‐9 enzymatic activity.

### 2.11 TUNEL apoptosis assay

Cells on the coverslips were fixed in 4% of paraformaldehyde for 30 min, followed by permeabilizing in 0.5% of Triton X‐100. Then, the coverslips were incubated with the TdT‐mediated dUTP nick end labeling (TUNEL) (Beyotime, Shanghai, China) for 2 hours. After incubation with DAPI for 5 min, the anti‐fade solution was added to the coverslips, followed by photography under the fluorescent microscope.

### 2.12 Transfection

The scrambled siRNA, Na_V_1.5 siRNAs, and FAK siRNAs (Supplemental Table S3) were synthesized by GenePharma (Shanghai, China). Cells reached in 70% confluence were transiently transfected with the siRNA using Lipofectamine 2000 reagent (Invitrogen) according to the manufacturer's instructions. The transfection reagent was removed 6 hours later, and the total protein, conditional medium and RNA was collected after 48 hours for further detection.

### 2.13 Tumorigenesis and metastasis assay *in vivo*

C57BL/6 female mice (4–6 weeks) (Laboratory Animal Center of Dalian Medical University) were maintained under controlled environmental conditions. To set up transcoelomic dissemination and metastasis model, murine syngeneic ovarian cancer (ID8) cells (1 × 10^7^, in 100 µl saline) were intraperitoneally (i.p.) injected to female mice, followed by lidocaine in saline (0.5%, 50 µl) i.p. injection once daily for 3 days. On the day 7, mice were randomly divided into four groups (n = 8/group) and received different i.p. injections: control (saline), lidocaine, cisplatin (60 μg kg^−1^), and a combination of lidocaine and cisplatin. The general status was monitored and body weight was recorded. B‐ultrasound apparatus (Vevo1100, WINSUN, Beijing, China) was used to monitor abdominal mass and ascites. Mice were sacrificed by cervical vertebra dislocation on the day 14, and tumor tissues were dissected for the analyses. All animal experimental procedures were in accordance with the guidelines of laboratory animals in Dalian Medical University and Peking University Third Hospital.

## 3 RESULTS

### 3.1 Lidocaine inhibits the proliferation of ovarian cancer cells

**Figure 1:**
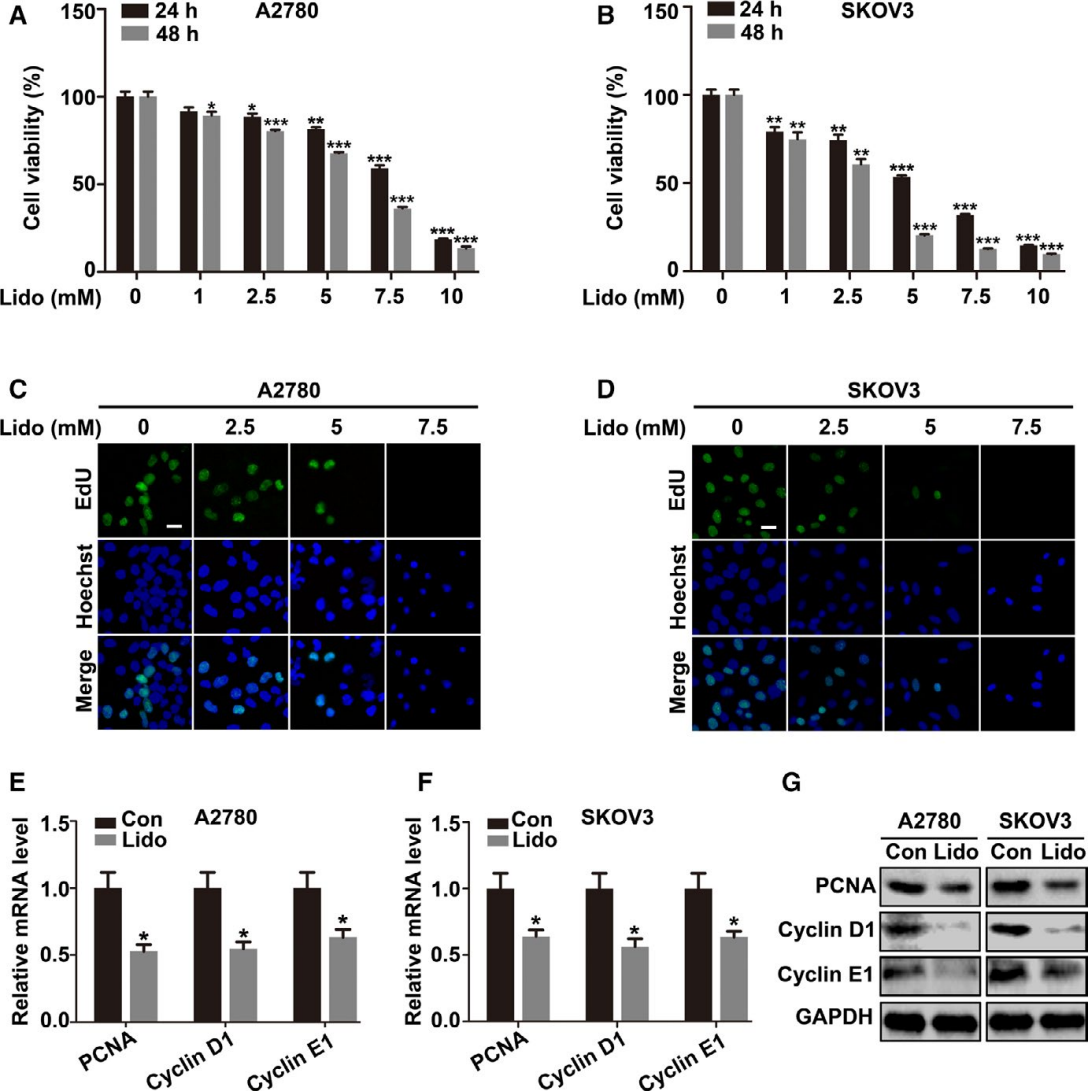
Lidocaine inhibits the proliferation of ovarian cancer cells. A and B, A2780 and SKOV3 cells were treated with lidocaine (0, 1, 2.5, 5, 7.5, and 10 mM) for 24 and 48 hrs, respectively. CCK‐8 assay was used for cell viability evaluation. C and D, A2780 and SKOV3 cells were exposed to lidocaine (0, 2.5, 5, and 7.5 mM) for 48 hrs. Representative images of FITC‐labeled EdU (green) incorporation assay were presented. Hoechst 33342 (blue) was used for nuclei staining. Bar represents 50 μm. E and F, qRT‐PCR and (G) Western blot showed the mRNA and protein expression levels of PCNA, Cyclin D1, and Cyclin E1 in control and lidocaine‐ (5 mM) treated cells. GAPDH was used as an internal control. The data were presented as mean ±SEM (n = 9); *p < 0.05, **p < 0.01, ***p < 0.001

To examine whether lidocaine exerts the antiproliferative effect on ovarian cancer cells, A2780 and SKOV3 cells were treated with lidocaine (0, 1, 2.5, 5, 7.5, and 10 mM) for 24 hours and 48 hours, respectively (Figure 1). Cell viability was reduced in a dose dependent manner with a significant reduction at 5 mM of lidocaine compared with the control (*p* < 0.01 and *p* < 0.001, respectively) in A2780 and SKOV3 cells for 24 hours and 48 hours, respectively (Figure 1A,B). The cell proliferation assessed by EdU incorporation assay showed that incorporated FITC‐labeled EdU (Green) in the nuclei of the cancer cells was decreased compared with the control after lidocaine treatment for 48 hours (Figure 1C,D). Furthermore, the levels of the proliferation‐related markers (PCNA, Cyclin D1, and Cyclin E1) detected by qRT‐PCR and western blot were also significantly decreased compared with the control (*p* < 0.05) (Figure 1E‐G).

### 3.2 Lidocaine inhibits EMT, migration, and invasion of ovarian cancer cells

**Figure 2:**
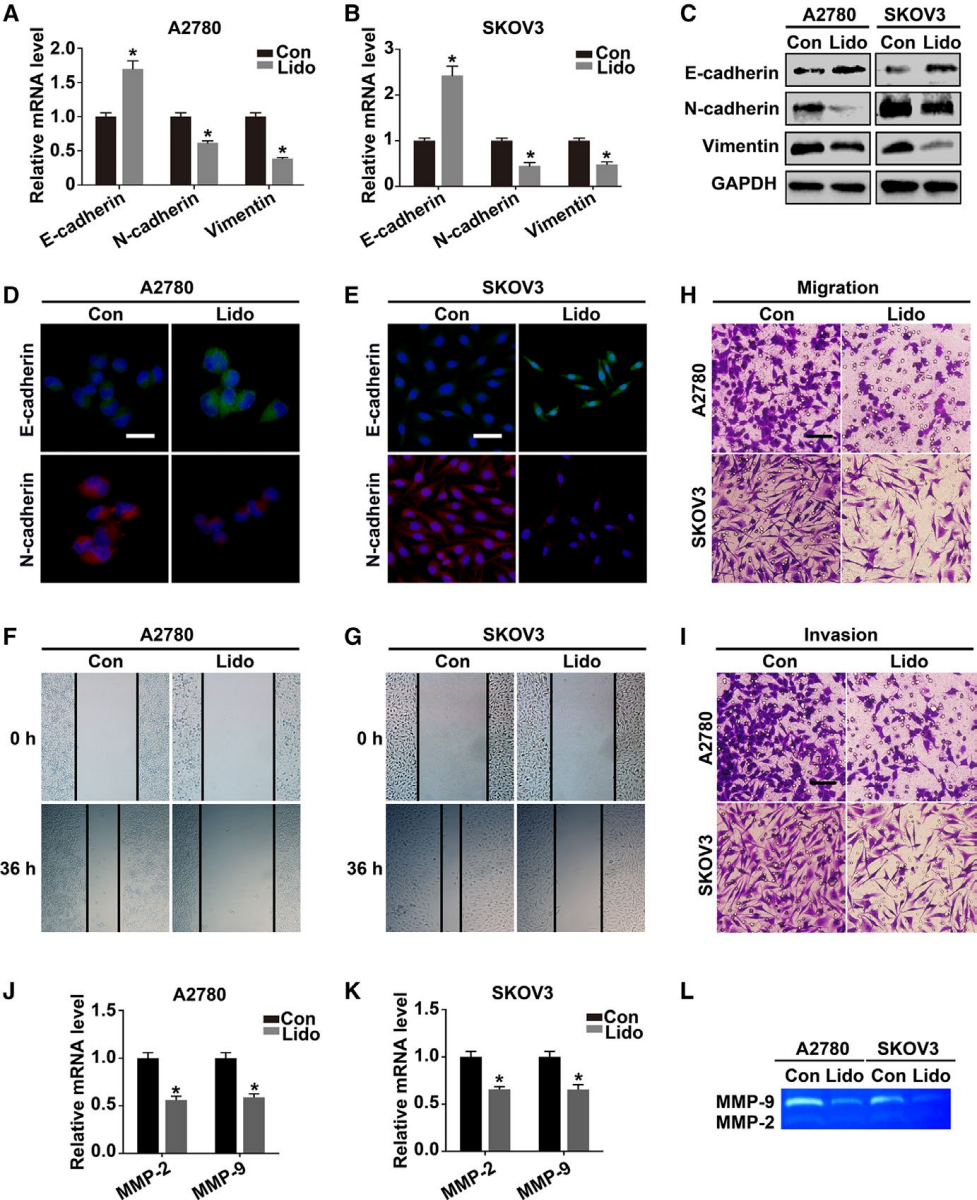
Lidocaine inhibits EMT, migration, and invasion of ovarian cancer cells. A and B, qRT‐PCR and (C) Western blot analysis of EMT markers (E‐cadherin, N‐cadherin, and Vimentin) in control and lidocaine‐treated A2780 and SKOV3 cells. GAPDH was used as an internal control. D and E, Representative fluorescent images of E‐cadherin and N‐cadherin in lidocaine‐treated A2780 and SKOV3 cells. DAPI (blue) was used for nuclei staining. F and G, Scratch assay, (H and I) Transwell migration and matrigel invasion assays were performed to detect the migration and invasion potential. J and K, qRT‐PCR and (L) gelatin zymography analysis of mRNA expression levels and enzymatic activity of MMP‐2 and MMP‐9 after lidocaine treatment in A2780 and SKOV3 cells. Bars represent 20 μm (D and E) and 100 μm (H and I). The data were presented as mean ±SEM (n = 3); *p < 0.05

Given that the characteristic high metastasis potential of ovarian cancer cells is closely related to clinical recurrence and progression, whether lidocaine could inhibit the metastasis potential was determined (Figure 2). The qRT‐PCR results showed that lidocaine significantly increased the level of epithelial marker E‐cadherin in both A2780 and SKOV3 cells; whereas decreased the levels of mesenchymal markers N‐cadherin and Vimentin compared with the controls (*p* < 0.05), respectively (Figure 2A,B). The accordant changes were also found by western blot analysis (Figure 2C). Immunofluorescent staining of E‐cadherin and N‐cadherin showed similar changes (Figure 2D,E). Scratch assay, transwell migration, and matrigel invasion assays revealed that lidocaine significantly restrained the motility capability of both cells (Figure 2F‐I). In addition, qRT‐PCR and gelatin zymography showed that lidocaine also decreased the mRNA expression and enzymatic activity of MMP‐2 and MMP‐9 (Figure 2J‐L).

### 3.3 Lidocaine sensitizes ovarian cancer cells to cisplatin *in vitro*

**Figure 3:**
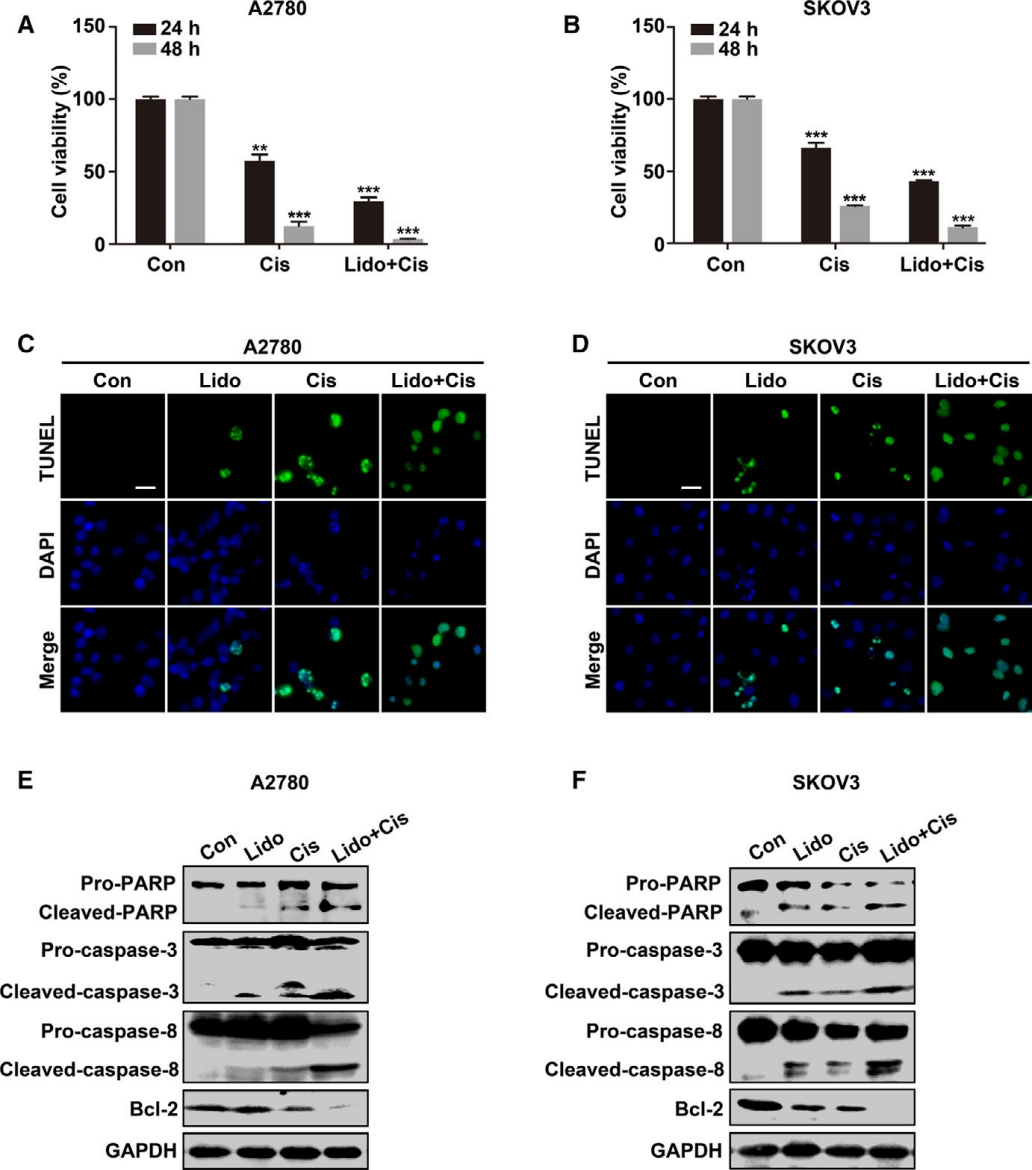
Lidocaine sensitizes ovarian cancer cells to cisplatin in vitro. A and B, A2780 and SKOV3 cells were untreated, treated with cisplatin (10 μM), or cisplatin combined with lidocaine (5 mM) for 24 and 48 hrs, respectively. Cell viability was evaluated by CCK‐8 assay. C and D, Representative images of TUNEL (green)‐labeled apoptotic A2780 and SKOV3 cells. DAPI (blue) was used for nuclei staining. E and F, Western blot analysis of apoptotic marker proteins in control, lidocaine, cisplatin, cisplatin, and lidocaine combination groups. GAPDH was used as an internal control. Bar represents 20 μm. The data were presented as mean ±SEM (n = 9, n = 3); **p < 0.01, ***p < 0.001

Whether lidocaine augments the chemotherapeutic efficacy of cisplatin which is a first‐line chemotherapeutic drug of ovarian cancer was assessed. Results of CCK‐8 assay showed that cisplatin (10 μM) combined with lidocaine (5 mM) decreased the cell viability stronger than cisplatin alone (*p* < 0.01 and *p* < 0.001, respectively) in both A2780 and SKOV3 cells after treatment for 24 hours and 48 hours (Figure 3A,B). TUNEL apoptosis assay and the analysis of apoptosis‐related proteins by western blot also showed that the apoptotic cells (green) were increased in the cells treated with the combination of cisplatin and lidocaine (Figure 3C,D). The higher expression of active apoptotic proteins (Cleaved‐PARP, Cleaved‐caspase‐3, and Cleaved‐caspase‐8); while the lower level of antiapoptotic protein Bcl‐2, were detected in lidocaine and cisplatin combination treatment (Figure 3E,F).

### 3.4 Downregulation of Na_V_1.5 expression decreases metastatic capability of ovarian cancer cells

**Figure 4:**
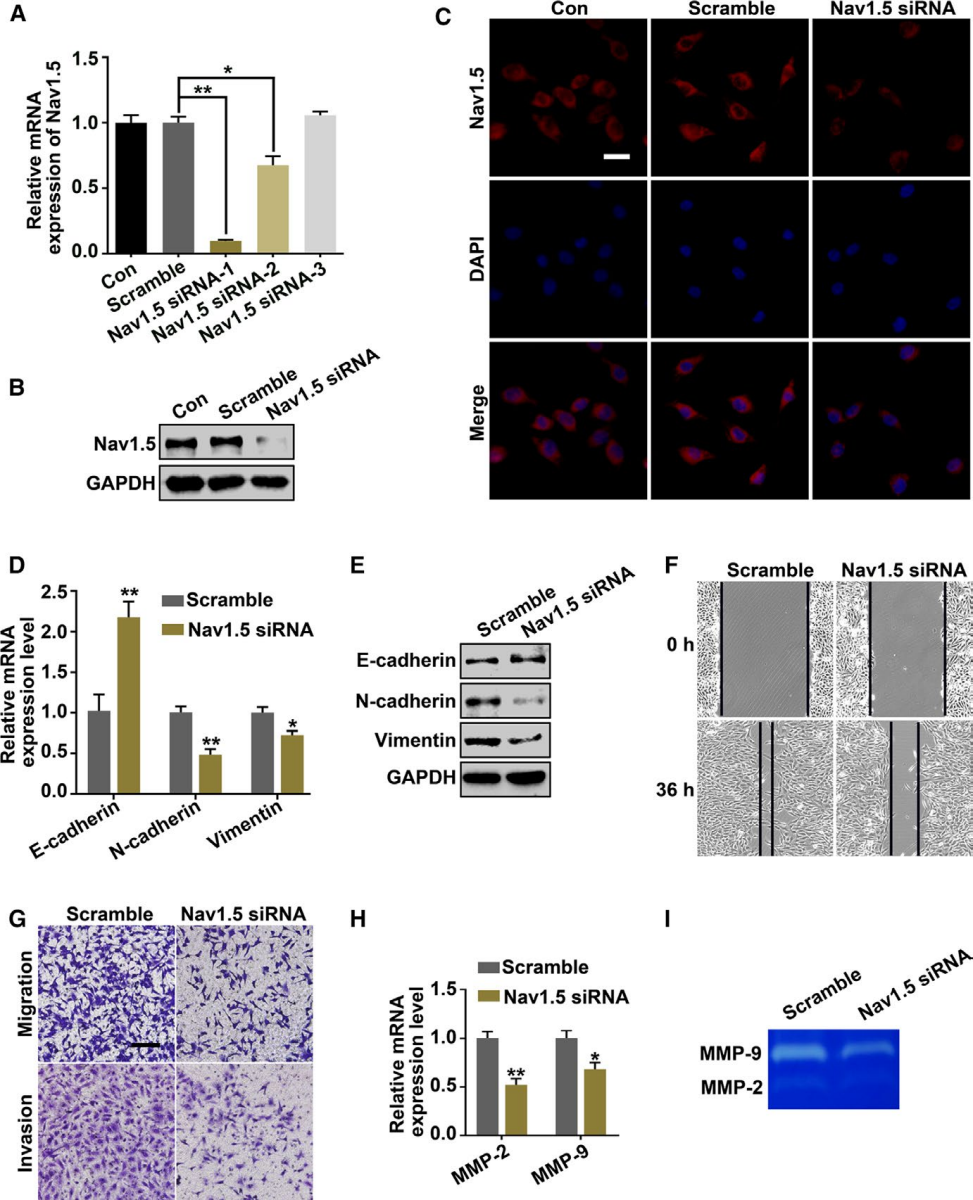
Downregulation of Na_V_1.5 expression suppresses the metastatic capability of ovarian cancer cells. A, qRT‐PCR, (B) Western blot, and (C) Immunofluorescent staining were used to detect the knockdown efficiency of Na_V_1.5 siRNAs (−1, −2, and −3) after transfection of SKOV3 cells. D, qRT‐PCR and (E) Western blot analysis of EMT markers (E‐cadherin, N‐cadherin, and Vimentin) in scrambled siRNA and Na_V_1.5 siRNA‐1 transfected cells. GAPDH was used as an internal control. F, Scratch assay, (G) Transwell migration and matrigel invasion assays were performed to detect the cellular motility of SKOV3 cells. H, qRT‐PCR and (I) Gelatin zymography showed the mRNA expression level and enzymatic activity of MMP‐2 and MMP‐9 after scramble siRNA and Na_V_1.5 siRNA‐1 transfection. Bars represent 20 μm (C) and 100 μm (G). Data were presented as mean ±SEM (n = 3); *p < 0.05, **p < 0.01

The association of Na_V_1.5 level and the metastatic potential of ovarian cancer cells was explored. We first demonstrated that Na_V_1.5 level was highly expressed in the human cancerous ovarian tissues (71 cases, stage II–III) compared to the normal ovarian tissues (30 cases), and the level was also higher in human ovarian metastatic lesions than those in the primary ovarian cancer tissues among the 16 paired samples by immunohistofluorescent analysis (Supplemental Figure S1). To further explore that whether Na_V_1.5 was involved in the migration and invasion of ovarian cancer cells, three siRNAs targeting Na_V_1.5 were transfected to SKOV3 cells. As shown in Figure 4A, Na_V_1.5 siRNA‐1 and −2 transfection significantly downregulated the gene expression of Na_V_1.5 compared with scramble siRNA transfection (*p* < 0.01 and *p* < 0.05, respectively), and was further confirmed by western blot and immunofluorescent staining (Figure 4B,C). The downregulation of Na_V_1.5 by Na_V_1.5 siRNA‐1 transfection inhibited EMT by increasing E‐cadherin (*p* < 0.01); while decreasing N‐cadherin (*p* < 0.01) and Vimentin levels (*p* < 0.05) by qRT‐PCR and western blot (Figure 4D,E). Meanwhile, the mobility capability of the cells was also reduced by scratch assay, transwell migration, and matrigel invasion assays (Figure 4F,G. The decreased gene expression and enzymatic activity of MMP‐2 and MMP‐9 by qRT‐PCR and gelatin zymography further confirmed the invasive and metastatic inhibition through downregulating Na_V_1.5 level (Figure 4H,I).

### 3.5 Lidocaine suppresses cancer cell malignancy and enhances cisplatin sensitivity by blocking Na_V_1.5‐mediated FAK/Paxillin signaling pathway

**Figure 5:**
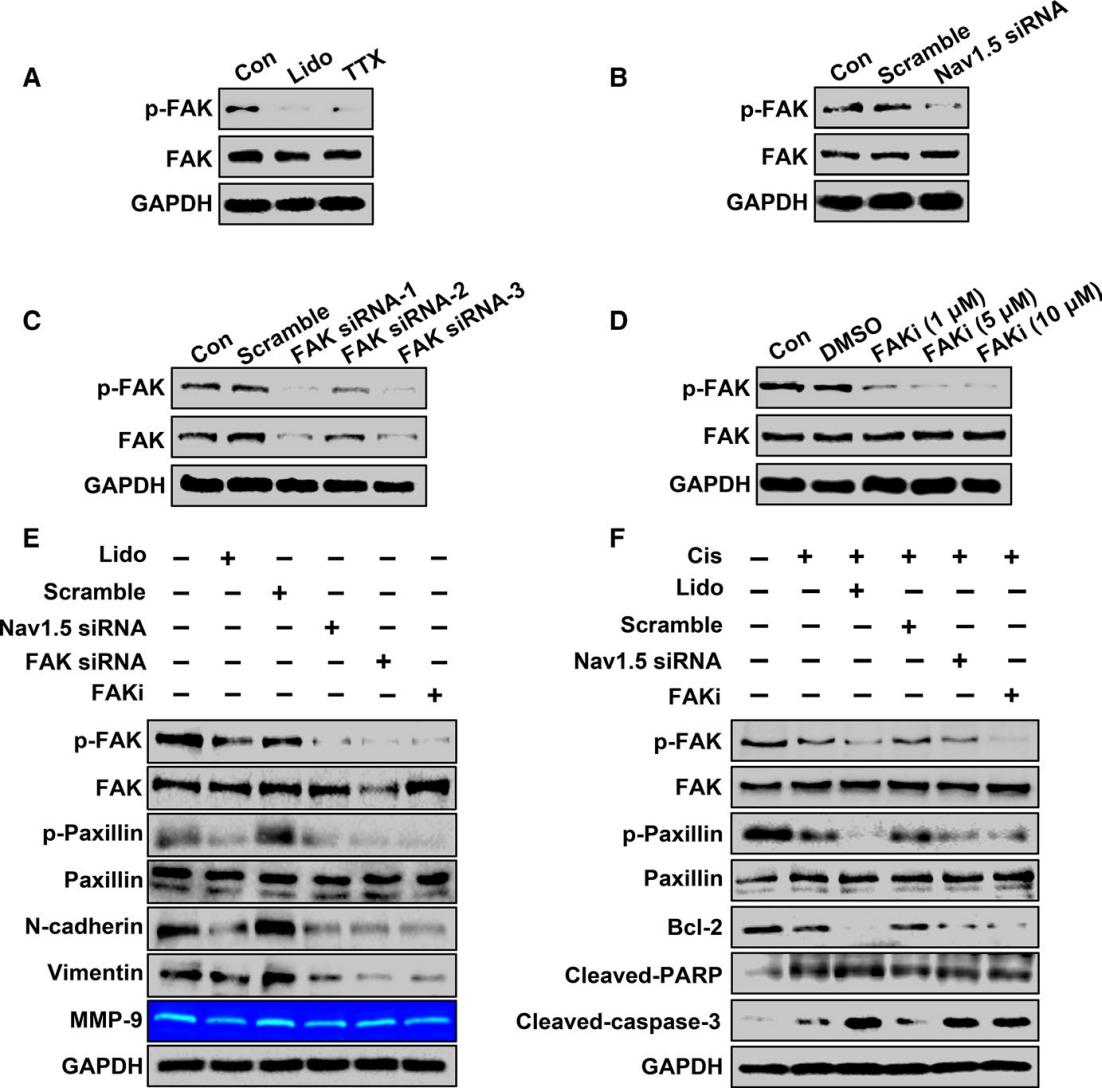
Lidocaine suppresses cancer cell malignancy and enhances the cisplatin sensitivity by blocking Na_V_1.5‐mediated FAK/paxillin signaling pathway in SKOV3 cells. A and B, Western blot analysis of p‐FAK and FAK in lidocaine and TTX‐treated cells, as well as in scramble RNA and Na_V_1.5 siRNA transfected cells, respectively. C and D, Western blot analysis of p‐FAK and FAK in scrambled siRNA, FAK siRNAs (−1, −2, and −3) transfected cells, or in DMSO and FAK inhibitor‐ (1 μM, 5 μM, and 10 μM) treated cells, respectively. E, SKOV3 cells were treated with lidocaine, or transfected with scrambled siRNA, Na_V_1.5 siRNA, FAK siRNA, as well as addition of FAK inhibitor, and the levels of p‐FAK, FAK, p‐Paxillin, Paxillin, N‐cadherin, and Vimentin were detected by western blot. The enzymatic activity of MMP‐9 was detected by gelatin zymography analysis. F, SKOV3 cells were treated with cisplatin, or in combination with lidocaine, scrambled siRNA, Na_V_1.5 siRNA, and FAK inhibitor. The levels of p‐FAK, FAK, p‐Paxillin, Paxillin, Bcl‐2, Cleaved‐caspase‐3, and Cleaved‐PARP were detected by western blot

The effects of lidocaine, tetrodotoxin (TTX, Absin Bioscience Inc., Shanghai, China), and Na_V_1.5 knockdown on the inhibition of FAK activation were assayed. Herein, TTX (50 μM) was used as a general blocker of VGSCs. As shown in Figure 5A,B, lidocaine, TTX, and downregulation of Na_V_1.5 expression significantly decreased p‐FAK level as compared to those in the controls and scramble siRNA transfection in SKOV3 cells. To further investigate the roles of FAK/Paxillin signaling pathway and the alterations of downstream signaling proteins in the inhibition of ovarian cancer cell transformation exerted by lidocaine, the reduced p‐FAK levels were determined by FAK siRNAs (−1, −2, and −3) transfection and addition of FAK inhibitor (FAKi) (PF‐562271, Selleck Chemicals, Huston, USA) in different concentrations (1, 5, and 10 μM) by western blot (Figure 5C,D), and FAK siRNA‐1 and FAKi (5 μM) were selected for further study. As shown in Figure 5E, lidocaine inhibited FAK/Paxillin activation, decreased N‐cadherin and Vimentin expression, and reduced MMP‐9 enzymatic activity, compared with the control (lane 2 vs.1). The accordant decrease induced by Na_V_1.5 siRNA and FAK siRNA transfection, as well as FAKi treatment was found. Furthermore, the combination of lidocaine and cisplatin strengthened the inactivation of FAK/Paxillin signaling pathway and induction of apoptosis in comparison with cisplatin alone (Figure 5F, lane 3 vs. 2).

### 3.6 Lidocaine inhibits tumorigenesis and metastasis of ovarian cancer *in vivo*

**Figure 6:**
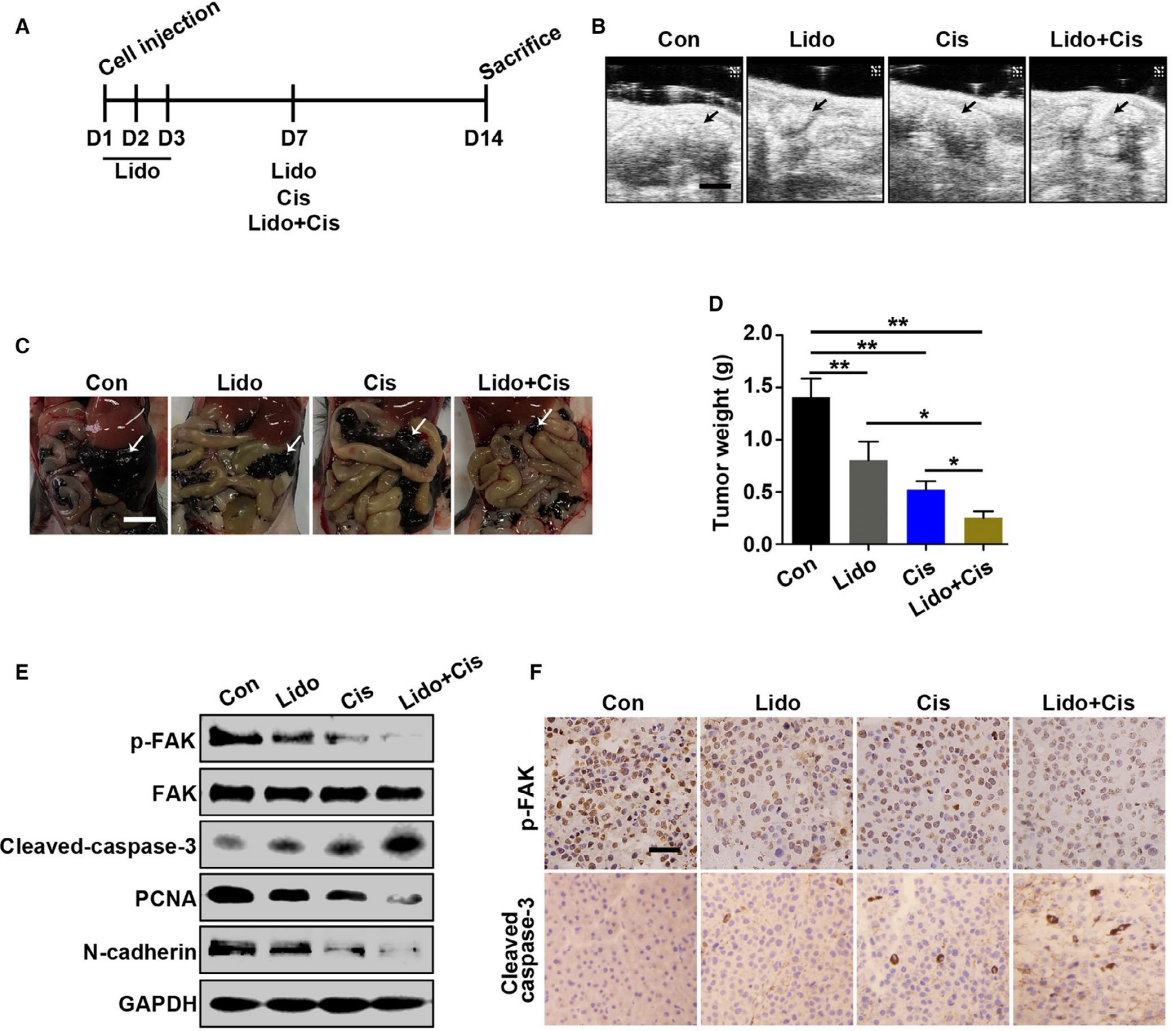
Lidocaine inhibits tumorigenesis and metastasis of ovarian cancer in vivo. A, Schematic representation of the treatment paradigm in this study. B, Representative photographs of the abdominal implantation metastasis foci of murine ovarian cancer in control (saline), lidocaine, cisplatin, or the combination of lidocaine and cisplatin group by B‐ultrasonography, respectively. Bar represents 20 μm. C, Representative pictures of the abdominal implantation metastasis foci in differently treated groups viewed after laparotomy, respectively. Bar represents 15 mm. D, Statistical analysis of the excised tumor weight in differently treated groups. E, Western blot analysis of p‐FAK, FAK, Cleaved‐caspase‐3, PCNA, and N‐cadherin of cancer tissues collected on the day 14. GAPDH was used as an internal control. F, Representative images of immunohistochemical staining of p‐FAK and Cleaved‐caspase‐3. Bar represents 50 μm. Data were presented as mean ±SEM (n = 8); *p < 0.05, **p < 0.01

The experiments were designed based on the fact that the drainage tube is normally retentioned for 3 days after operation, which can be used for intraperitoneal drug administration, and lidocaine abdominal administration reduced VAS score and opioid drug use postoperation.^17^ After murine syngeneic ovarian cancer (ID8) cells were injected into the abdominal cavity to simulate the spread of the cancer cells caused by operation, lidocaine, or in combination with cisplatin i.p. delivered to observe the inhibitory effects on the intraperitoneal proliferation and metastasis of ovarian cancer cells. Herein, cisplatin was administered on the day 7 to imitate the initiation of cisplatin chemotherapy till on the day 14 end point (Figure 6A). The general status (nutrition, movement, mental, and fur, etc.) showed no obvious difference, and body weight was not significantly changed (*p* > 0.05) among the groups. The transcoelomic dissemination and metastasis model was successfully established by B‐ultrasonography (Figure 6B), and bloody ascites and multiple metastatic foci (white color arrows) mainly on enteromesenterium were found after laparotomy (Figure 6C), indicating the similarity of murine ovarian cancer model with human ovarian cancer of advanced and late stage. The results also showed that lidocaine, cisplatin, and the combination of lidocaine and cisplatin decreased ovarian cancer loading (weight of excised metastatic foci) compared to the control (*p* < 0.05 and *p* < 0.01, respectively) (Figure 6D). Meantime, the data manifested that the combination application was more efficient in inhibiting cancer malignancy than single drug usage. Furthermore, p‐FAK, PCNA, and N‐cadherin levels were decreased; while Cleaved‐caspase‐3 increased by western blot (Figure 6E). The immunohistochemical staining of p‐FAK and Cleaved‐caspase‐3 present the accordant alterations (Figure 6F).

## 4 DISCUSSION

**Figure 7:**
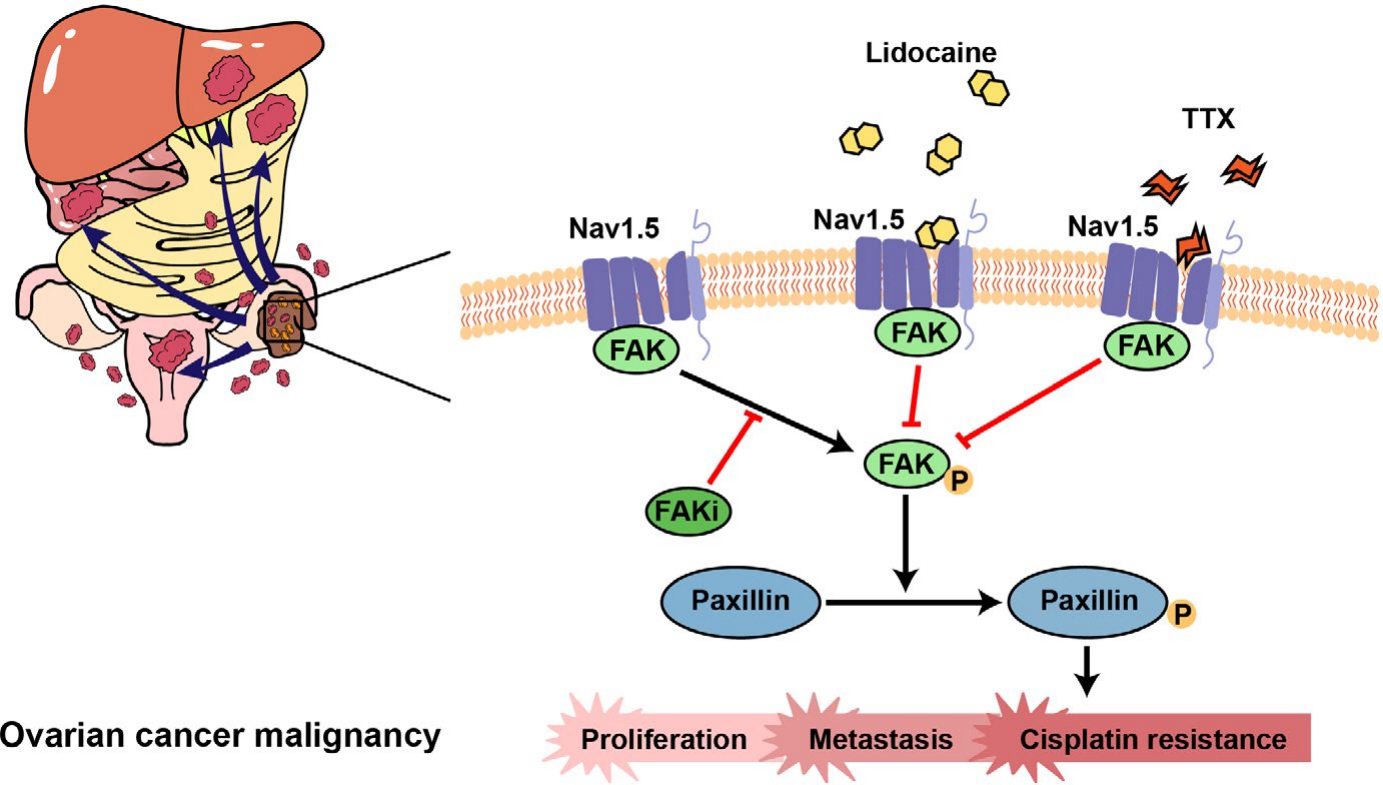
Schematic representation of the molecular mechanism in the suppression of ovarian cancer malignancy by lidocaine. Ovarian cancer cells expressing high Na_V_1.5 level present strong capacity of intraperitoneally implanted metastasis. Lidocaine binding with Na_V_1.5 blocks the activation of FAK/Paxillin signaling pathway, inhibits the proliferation and metastasis, and decreases cisplatin resistance of ovarian cancer cells. The voltage‐gated sodium channels (VGSCs) blocker, tetrodotoxin (TTX), or FAK inhibitor (FAKi) reduces ovarian cancer malignancy by inactivating FAK/Paxillin signaling pathway

This study provided the evidence that lidocaine inhibited the proliferation, EMT, and metastasis, as well as induced the apoptosis in both ovarian cancer cells and ovarian cancer tissues of a murine syngeneic ovarian cancer model. The cisplatin sensitivity was also enhanced by lidocaine. The analysis of human ovarian cancer tissues revealed the association between Na_V_1.5 level and metastasis potential of ovarian cancer. Further investigations indicated that blocking Na_V_1.5 by lidocaine or sodium ion channel blocker decreased the ovarian malignancy through inactivation of FAK/Paxillin signaling pathway (Figure 7).

The Na_V_1.5 level is highly expressed in breast and colon cancer.^15^, ^16^ A previous study reported that a high Na_V_1.5 expression was correlated with high malignancy and metastasis of ovarian cancer.^18^ We found that metastatic ovarian cancer tissues present the higher level of Na_V_1.5 than those of the in situ “mother” cancer or normal ovarian tissues (Figure S1). Particularly, EMT is correlated with the metastasis of ovarian cancer,^19^ and Na_V_1.5 knockdown inhibited EMT and impaired the motility and invasion capability of ovarian cancer cells shown in our study. Taken together, Na_V_1.5 likely drives the malignant transformation and metastasis of ovarian cancer. In addition, the local anesthetics (lidocaine, levobupivacaine, and ropivacaine) act through Na_V_1.5 blockage to inhibit proliferation and metastasis potential.^20^, ^21^ Retrospective studies showed that using amide local anesthetics for regional anesthesia during perioperative period reduced the recurrence rate of the cancer patients.^22^, ^23^ Xuan et al found that bupivacaine inhibited the proliferation of ovarian cancer cells.^24^ Lidocaine has also been reported to inhibit the invasion of lung cancer and attenuate the metastasis of breast cancer.^25^, ^26^ In our study, the ovarian cancer cells were treated with lidocaine; at its concentration of 5 mM, the proliferation and metastasis potential of ovarian cancer was significantly suppressed and its effects, at least in part, was through its inhibition of Na_V_1.5.

The FAK level was greatly increased in the late stage ovarian cancers, indicating the strong association of FAK with high metastasis and recurrence characteristics of ovarian cancer. FAK is also linked to the higher pathological stage, metastasis, and shorter overall survival rate, as well as drug resistance to platinum‐ and taxane‐based chemotherapy of ovarian cancer patients.^27^, ^28^ The recent successes in using small molecule FAK inhibitors in the clinical trials manifest the significance and function of FAK in ovarian cancer biology. ^29^ At clinically relevant concentrations, lidocaine was found to inhibit the angiogenesis of endothelial cells by inducing apoptosis and inactivating FAK/Paxillin signal pathway.^30^ Piegeler et al also reported that lidocaine and ropivacaine suppressed TNFα‐induced invasion of lung adenocarcinoma cells by inhibition of Akt and FAK activation.^25^ Our study showed that lidocaine, Na_V_1.5 siRNA transfection, and VGSCs blocker TTX significantly decreased FAK/Paxillin activation, and inhibited EMT and metastasis capability both *in vitro* and *in vivo*. The underlying mechanism of lidocaine/Na_V_1.5/FAK axis may be related to the direct binding of cytoplasmic region in Na_V_1.5 α‐subunit with FAK, and thus, dephosphorylating FAK‐mediated signaling pathway.

The strong metastatic potential of ovarian cancer cells facilitates them to disseminate into the adjunctive tissues and organs, such as liver and omentum majus, in the abdominopelvic cavity.^2^ The intraperitoneal delivery of chemotherapeutics is effective in the treatment of abdominal metastatic tumors. One of its advantages is that the drug can directly interfere with the residual or micrometastatic cancer cells, as well as the spreading cancer cells during the operation.^31^ According to NCCN clinical practice guidelines in ovarian cancer (2018), II–IV ovarian patients are recommended for postoperative intraperitoneal platinum chemotherapy.^32^ Intraperitoneal application of lidocaine in the patients underwent gynecology surgery reduced VAS score and consumption of opioids.^17^, ^33^, ^34^ Lidocaine by i.p. injection in our study of an in vivo ovarian cancer model also showed its antimetastatic effects. The multi‐effects of lidocaine, such as analgesia, antiproliferation, antimetastasis, cisplatin sensitivity enhancement indicate that its intraperitoneal application during ovarian cancer surgery should be considered although subjected further study in clinically setting.

The choice of anesthetic techniques and anesthetics may affect the outcome of cancer patients.^35^ It has been reported that general anesthetics, such as isoflurane, promote the growth and migration capability of renal, and ovarian cancer cells.^36^, ^37^ Furthermore, at a clinical relevant concentration, isoflurane, sevoflurane, and desflurane promoted ovarian cancer metastasis.^38^ Giving that the local anesthetic application is sparing opioids use during perioperative period, and hence, reduces its side effects, our findings may highlight that local anesthetic use during perioperative period may be beneficial to the cancer patients although warrants further clinical study.

Our work is not without experimental limitations. The concentration of lidocaine used in our *in vitro* experiments may be high than clinical use. However, the highest concentration (10 mM) used is equal to 0.24% which is far lower than usually clinical local used concentration of 2% while in in vivo part, its concentration was used only at 0.5%. Therefore, although one can argue that this study is a proof of concept study and is not at the best clinical reality, the translational value of our study is still considerably high for its direct abdominal local use.

Collectively, our work showed that Na_V_1.5 was highly expressed by the metastatic lesion as relative to the primary ovarian cancer and normal tissues. The results also indicated that lidocaine inhibited EMT and metastasis potential of ovarian cancer cells, and Na_V_1.5 knockdown or blockage impaired the EMT and metastatic properties of ovarian cancer cells. The data manifest that lidocaine suppresses the cellular metastatic potential and enhances the cisplatin sensitivity by blocking Na_V_1.5/FAK/Paxillin signaling pathway. The clinical value for lidocaine directly applied into the abdominal cavity during ovarian cancer surgery is needed to assess urgently.

## CONFLICT OF INTEREST

The authors have no conflict of interests.

## AUTHOR CONTRIBUTIONS

Study/design/planning, C.L., H.Y.G., D.M., X.Y.G.; Study conduct, C.L., M.Y., H.W.; Data analysis, C.L., Y.L., C.Y.X., X.Q.Z.; Writing paper, C.L., D.M., X.Y.G., M.L.; Revising paper, all authors. All authors have approved to submit for a publication.

## Supporting information

Fig S1

Table S1

Table S2

Table S3

Supplementary Material

## Data Availability

Data are available from the corresponding author (X.Y.G.) upon reasonable request.
