# Supplementary figures and images for: Lidocaine inhibits the metastatic potential of ovarian cancer by blocking NaV1.5‐mediated EMT and FAK/Paxillin signaling pathway
Source: Cancer Med. 2020 Dec 6;10(1):337–49. doi: 10.1002/cam4.3621 (PMC7826465; doi:10.1002/cam4.3621)

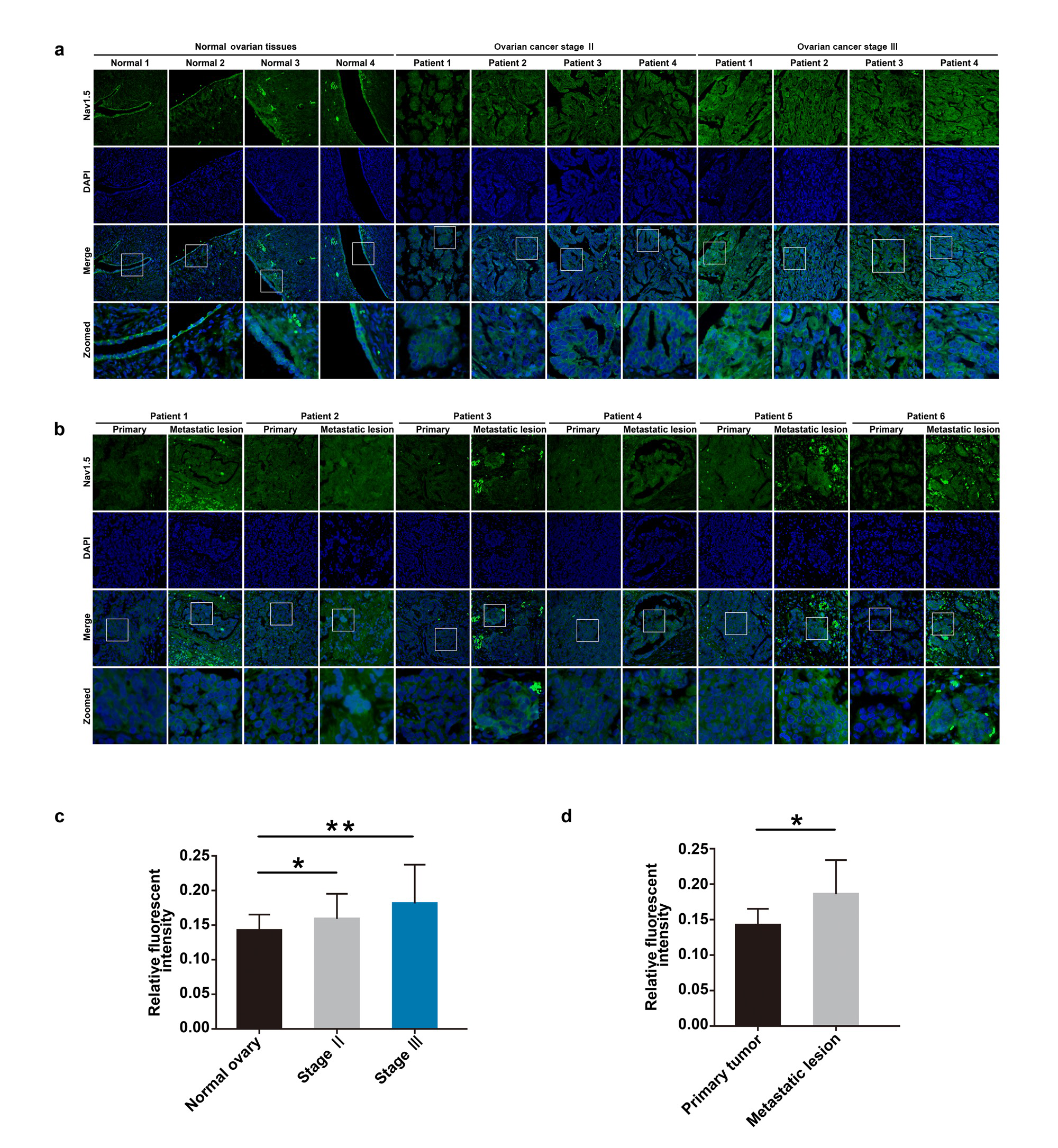

Supplement: Supplementary file 1 — Fig S1 [file CAM4-10-337-s001.tif]
