# Supplementary material for: Lidocaine inhibits the metastatic potential of ovarian cancer by blocking NaV1.5‐mediated EMT and FAK/Paxillin signaling pathway
Source: Cancer Med. 2020 Dec 6;10(1):337–49. doi: 10.1002/cam4.3621 (PMC7826465; doi:10.1002/cam4.3621)
Supplement: Supplementary file 2 — Table S1 [file CAM4-10-337-s002.docx]

**Table S1 Clinical data of human ovarian tissues**

| **Normal ovarian tissues** **(OV806)** | | | **Cancerous ovarian tissues**  **(OV8010)** | | | | | | | | | | **Paired tissues** | | | | |
| --- | --- | --- | --- | --- | --- | --- | --- | --- | --- | --- | --- | --- | --- | --- | --- | --- | --- |
| **No.** | **Age** | **Pathology diagnosis** | **No.** | **Age** | **Pathology diagnosis** | **Stage** | **Type** | **No.** | **Age** | **Pathology diagnosis** | **Stage** | **Type** | **No.** | **Age** | **Pathology diagnosis** | **Stage** | **Type** |
| 1 | 50 | Normal | 1 | 69 | SPA | IIb | Malignant | 37 | 50 | SPA | IIIc | Malignant | 1 | 65 | SA | IIIc | Primary/Metastatic |
| 2 | 50 | Normal | 2 | 73 | SPA | IIa | Malignant | 38 | 54 | SPA | IIIc | Malignant | 2 | 44 | SA | IIIc | Primary/Metastatic |
| 3 | 63 | Normal | 3 | 60 | SPA | IIa | Malignant | 39 | 51 | SPA | IIIc | Malignant | 3 | 65 | SA | IV | Primary/Metastatic |
| 4 | 63 | Normal | 4 | 56 | SPA | II | Malignant | 40 | 58 | SPA | IIIc | Malignant | 4 | 63 | SA | IIIc | Primary/Metastatic |
| 5 | 39 | Normal | 5 | 35 | SPA | II | Malignant | 41 | 48 | SPA | IIIc | Malignant | 5 | 58 | SA | IIIc | Primary/Metastatic |
| 6 | 39 | Normal | 6 | 49 | SPA | IIc | Malignant | 42 | 38 | SPA | IIIc | Malignant | 6 | 60 | SA | IIa | Primary/Metastatic |
| 7 | 29 | Normal | 7 | 63 | SPA | IIb | Malignant | 43 | 57 | SPA | IIIc | Malignant | 7 | 49 | SA | IV | Primary/Metastatic |
| 8 | 29 | Normal | 8 | 49 | SPA | II | Malignant | 44 | 53 | SPA | IIIc | Malignant | 8 | 57 | SPA | IIIc | Primary/Metastatic |
| 9 | 40 | Normal | 9 | 60 | SPA | IIb | Malignant | 45 | 62 | SPA | III | Malignant | 9 | 54 | SA | IIIb | Primary/Metastatic |
| 10 | 40 | Normal | 10 | 45 | SPA | II | Malignant | 46 | 49 | SPA | IIIc | Malignant | 10 | 44 | SA | IIIb | Primary/Metastatic |
| 11 | 48 | Normal | 11 | 51 | SPA | IIa | Malignant | 47 | 65 | SPA | IIIc | Malignant | 11 | 46 | SA | IIIb | Primary/Metastatic |
| 12 | 48 | Normal | 12 | 65 | SPA | II | Malignant | 48 | 47 | SPA | IIIc | Malignant | 12 | 53 | SA | IV | Primary/Metastatic |
| 13 | 63 | Normal | 13 | 56 | SPA | II | Malignant | 49 | 42 | SPA | IIIc | Malignant | 13 | 58 | SA | IIIc | Primary/Metastatic |
| 14 | 63 | Normal | 14 | 41 | SA | IIc | Malignant | 50 | 46 | SPA | IIIc | Malignant | 14 | 52 | SA | IIIc | Primary/Metastatic |
| 15 | 36 | Normal | 15 | 56 | SA | II | Malignant | 51 | 56 | SPA | IIIc | Malignant | 15 | 65 | SPA | IIIb | Primary/Metastatic |
| 16 | 36 | Normal | 16 | 39 | SA(n) | II | Malignant | 52 | 56 | SPA | IIIc | Malignant | 16 | 46 | SA | IIIc | Primary/Metastatic |
| 17 | 45 | Normal | 17 | 48 | SA | IIa | Malignant | 53 | 42 | SPA | IIIc | Malignant |  |  |  |  |  |
| 18 | 45 | Normal | 18 | 75 | SA | II | Malignant | 54 | 32 | SPA | IIIc | Malignant |  |  |  |  |  |
| 19 | 45 | Normal | 19 | 43 | SA | II | Malignant | 55 | 46 | SPA | IIIc | Malignant |  |  |  |  |  |
| 20 | 45 | Normal | 20 | 51 | SA | IIa | Malignant | 56 | 26 | SPA | IIIc | Malignant |  |  |  |  |  |
| 21 | 53 | Normal | 21 | 45 | SA | IIb | Malignant | 57 | 57 | SPA | IIIc | Malignant |  |  |  |  |  |
| 22 | 53 | Normal | 22 | 59 | SA | II | Malignant | 58 | 43 | MPA | IIIc | Malignant |  |  |  |  |  |
| 23 | 42 | Normal | 23 | 48 | SA | IIb | Malignant | 59 | 48 | MA | IIIc | Malignant |  |  |  |  |  |
| 24 | 42 | Normal | 24 | 41 | MA | IIa | Malignant | 60 | 52 | TCC | IIIc | Malignant |  |  |  |  |  |
| 25 | 40 | Normal | 25 | 40 | MPA | II | Malignant | 61 | 55 | TCC | IIIc | Malignant |  |  |  |  |  |
| 26 | 40 | Normal | 26 | 62 | A(s) | IIa | Malignant | 63 | 69 | SPA | II | Malignant |  |  |  |  |  |
| 27 | 48 | Normal | 27 | 50 | MA | II | Malignant | 64 | 37 | SPA | IIIa | Malignant |  |  |  |  |  |
| 28 | 48 | Normal | 28 | 41 | MA | IIa | Malignant | 65 | 65 | SPA | IIa | Malignant |  |  |  |  |  |
| 29 | 38 | Normal | 29 | 60 | EA(sn) | II | Malignant | 66 | 54 | SPA | IIa | Malignant |  |  |  |  |  |
| 30 | 38 | Normal | 30 | 50 | EA | IIa | Malignant | 68 | 56 | SPA | III | Malignant |  |  |  |  |  |
|  |  |  | 31 | 68 | EA | IIc | Malignant | 69 | 56 | SPA | IIIc | Malignant |  |  |  |  |  |
|  |  |  | 32 | 29 | A | IIa | Malignant | 70 | 48 | SA | IIIc | Malignant |  |  |  |  |  |
|  |  |  | 33 | 47 | MA | IIa | Malignant | 71 | 43 | SA | II | Malignant |  |  |  |  |  |
|  |  |  | 34 | 47 | CCC | IIa | Malignant | 73 | 41 | SA | IIa | Malignant |  |  |  |  |  |
|  |  |  | 35 | 66 | SA | IIIc | Malignant | 75 | 59 | EA | IIb | Malignant |  |  |  |  |  |
|  |  |  | 36 | 52 | SPA | IIIc | Malignant |  |  |  |  |  |  |  |  |  |  |

SPA: Serous papillary adenocarcinoma

SA: Serous adenocarcinoma

SA(n): Serous adenocarcinoma with necrosis

MA: Mucinous adenocarcinoma

A(s): Adenocarcinoma (sparse)

EA: Endometrioid adenocarcinoma

EA(sn): Endometrioid adenocarcinoma (sparse) with necrosis

A: Adenocarcinoma

CCC: Clear cell carcinoma

MPA: Mucinous papillary adenocarcinoma

TCC: Transitional cell carcinoma
