# Supplementary material for: Lidocaine inhibits the metastatic potential of ovarian cancer by blocking NaV1.5‐mediated EMT and FAK/Paxillin signaling pathway
Source: Cancer Med. 2020 Dec 6;10(1):337–49. doi: 10.1002/cam4.3621 (PMC7826465; doi:10.1002/cam4.3621)
Supplement: Supplementary file 3 — Table S2 [file CAM4-10-337-s003.docx]

**Table S2 Primers of genes** **for qRT-PCR**

| **Gene** | **Primers** |
| --- | --- |
| *Na_V_1.5* | 5'-CATGGCCAACTTCGCTTATGT-3', 5'-CGGCCCAGTGTTGAGGAT-3' |
| *PCNA* | 5'-TAAAGAAGAGGAGGCGGTAA-3', 5'-TAAGTGTCCCATGTCAGCAA-3' |
| *Cyclin D1* | 5'-TGTCCTACTACCGCCTCACA-3', 5'-CTTGGGGTCCATGTTCTGCT-3' |
| *Cyclin E1* | 5'-TGCAGCCAAACTTGAGGAAATC-3', 5'-TAGTCAGGGGACTTAAACGCCA-3' |
| *Vimentin* | 5'-CGTCTCTGGCACGTCTTGAC-3', 5'-GCTTGGAAACATCCACATCGA-3' |
| *N-cadherin* | 5'-AAAGAACGCCAGGCCAAAC-3', 5'-GGCATCAGGCTCCACAGTGT-3' |
| *E-cadherin* | 5'-CAACGACCCAACCCAAGAA-3', 5'-CCGAAGAAACAGCAAGAGCA-3' |
| *MMP-2* | 5'-TGATCTTGACCAGAATACCATCGA-3', 5'-GGCTTGCGAGGGAAGAAGTT-3' |
| *MMP-9* | 5'-CCTGGAGACCTGAGAACCAATC-3', 5'-CCACCCGAGTGTAACCATAGC-3' |
| *GAPDH* | 5'-GCACCGTCAAGGCTGAGAAC-3', 5'-TGGTGAAGACGCCAGTGGA-3' |
