# Supplementary material for: Lidocaine inhibits the metastatic potential of ovarian cancer by blocking NaV1.5‐mediated EMT and FAK/Paxillin signaling pathway
Source: Cancer Med. 2020 Dec 6;10(1):337–49. doi: 10.1002/cam4.3621 (PMC7826465; doi:10.1002/cam4.3621)
Supplement: Supplementary file 4 — Table S3 [file CAM4-10-337-s004.docx]

**Table S3 Sequences of siRNAs**

| **siRNA** | **Sequences** |
| --- | --- |
| *Scramble siRNA* | 5'-UUCUCCGAACGUGUCACGUTT-3', 5'-ACGUGACACGUUCGGAGAATT-3' |
| *Na_V_1.5 siRNA-1* | 5'-GCACAUGAUGGACUUCUUUTT-3', 5'-AAAGAAGUCCAUCAUGUGCTT-3' |
| *Na_V_1.5 siRNA-2* | 5'-GCAGGUGGCAACAUUUAAATT-3', 5'-UUUAAAUGUUGCCACCUGCTT-3' |
| *Na_V_1.5 siRNA-3* | 5'-GCAACGCUCUUUGAAGCAUTT-3', 5'-AUGCUUCAAAGAGCGUUGCTT-3' |
| *FAK siRNA-1* | 5'-CCCAGGUUUACUGAACUUATT-3', 5'-UAAGUUCAGUAAACCUGGGTT-3' |
| *FAK siRNA-2* | 5'-GCAAUGGAGCGAGUAUUAATT-3', 5'-UUAAUACUCGCUCCAUUGCTT-3' |
| *FAK siRNA-3* | 5'-CAGGUGAAGAGCGAUUAUATT-3', 5'-UAUAAUCGCUCUUCACCUGTT-3' |
