# Supplementary material for: Lidocaine inhibits the metastatic potential of ovarian cancer by blocking NaV1.5‐mediated EMT and FAK/Paxillin signaling pathway
Source: Cancer Med. 2020 Dec 6;10(1):337–49. doi: 10.1002/cam4.3621 (PMC7826465; doi:10.1002/cam4.3621)
Supplement: Supplementary file 5 — Supplementary Material [file CAM4-10-337-s005.docx]

**Fig. S1 Na_V_1.5 expression is high in the metastatic ovarian cancer tissues.** **(a)** Immunohistofluorescent analysis of Na_V_1.5 level in cancerous ovarian tissues (71 cases, stage II-III) in comparison with that in normal ovarian tissues (30 cases). Four representative pictures from normal ovarian tissues, stage II and stage III cancerous ovarian tissues, respectively. **(b)** Immunohistofluorescent analysis of Na_V_1.5 level in paired patient samples of both primary ovarian cancer and metastatic lesions (16 cases). Six representative pictures from the paired tissues. **(c)** Statistical analysis of relative fluorescent intensity of cancerous ovarian tissues *vs* normal ovarian tissues. **(d)** Statistical analysis of relative fluorescent intensity of metastatic lesions *vs* primary ovarian tissues. Bar represents 50 μm. Data were presented as mean ± SEM **p*<0.05, ***p*<0.01.
